# Verminous Aneurysm: A Rare Case

**DOI:** 10.7759/cureus.118674

**Published:** 2026-10-27

**Authors:** Omaima Taie, Amira Benjelloun, Michel Henry, Mustafa Taberkant, Hassan Chtata, Asmaa Mikou, Claude Laurian

**Affiliations:** 1 Vascular Surgery, Mohammed V Military Training Hospital, Rabat, MAR; 2 Vascular Surgery, Medexc Clinique, Hôpital Universitaire de Proximité Medexc de Rabat, Rabat, MAR; 3 Vascular Surgery, Clinique Louis Pasteur, Essey-lès-Nancy, FRA; 4 Anatomy and Cytopathology, Center for Anatomy and Cytopathology, Temara, MAR

**Keywords:** aneurysm, embolization, facial region, filariasis, surgery

## Abstract

Verminous aneurysm is an exceptionally rare vascular manifestation potentially associated with parasitic infection, particularly when involving unusual anatomical locations such as the facial region. We report the case of a 30-year-old man presenting with a facial vascular lesion in the setting of a previous history of filarial infection. This exceptional case highlights the diagnostic challenges associated with suspected filarial involvement in unusual vascular lesions. Surgical management involved direct exposure and resection of the vascular lesion, with a satisfactory postoperative clinical outcome. This case contributes to the medical literature by offering a new perspective on the management of rare vascular lesions with suspected filarial involvement, particularly in unusual anatomical locations, and emphasizes the diagnostic challenges and individualized management of such vascular manifestations. The rarity of this presentation and its clinical management provide valuable insights for both research and medical practice.

## Introduction

Verminous aneurysm is a rare subtype of aneurysm of parasitic origin. Although infectious aneurysms are most commonly associated with bacterial or fungal infections, the involvement of filariasis in their pathogenesis remains exceptionally rare. To date, verminous aneurysms have been very rarely reported in the literature [1]. Our case represents a very rare presentation of a facial vascular lesion with angiographic findings suggestive of filarial involvement, adding a novel element to this poorly understood condition.

Lymphatic filariasis is a parasitic disease caused by nematodes of the genera *Wuchereria* and *Brugia*, transmitted by mosquitoes. This infection induces a chronic inflammatory response and lymphatic obstruction, which can progress to elephantiasis [2]. However, the potential association between filarial infection and aneurysm formation remains poorly understood, with evidence limited to rare reported cases [1].

Verminous aneurysms have been described in horses, resulting from the migration of *Strongylus vulgaris* larvae and primarily affecting the mesenteric arteries [3]. This veterinary entity is distinct from the human filarial vascular manifestations discussed in the present case.

The significance of this case lies in the documentation of a rare vascular lesion in an unusual anatomical location, thereby contributing to the limited literature on this poorly understood condition. By highlighting its clinical manifestations, potential pathophysiological mechanisms, and therapeutic approaches, this case aims to enhance the understanding and management of rare vascular lesions with possible parasitic involvement.

## Case presentation

We report the case of a 30-year-old male from Gabon who presented to our clinic in Rabat, Morocco, with two non-inflammatory masses: one located on the left upper lip and the other on the left cheek and temporal region (Figure 1). The upper-lip lesion had been present since approximately 2012 and progressively increased in size, becoming cosmetically bothersome by 2015. Clinical examination revealed a palpable thrill and an audible bruit over both lesions. The upper-lip lesion measured approximately 25 × 10 mm and was located on the left upper lip, while the lesion involving the left cheek and temporal region measured approximately 4 × 4 cm. It was located in the anterior subpalpebral region and was mobile over the deeper planes, involving the subcutaneous tissues overlying the muscles attached to the maxilla. The lesion was supplied by branches of the maxillary and facial arteries, with two vascular pedicles and associated venous drainage channels.

**Figure 1:**
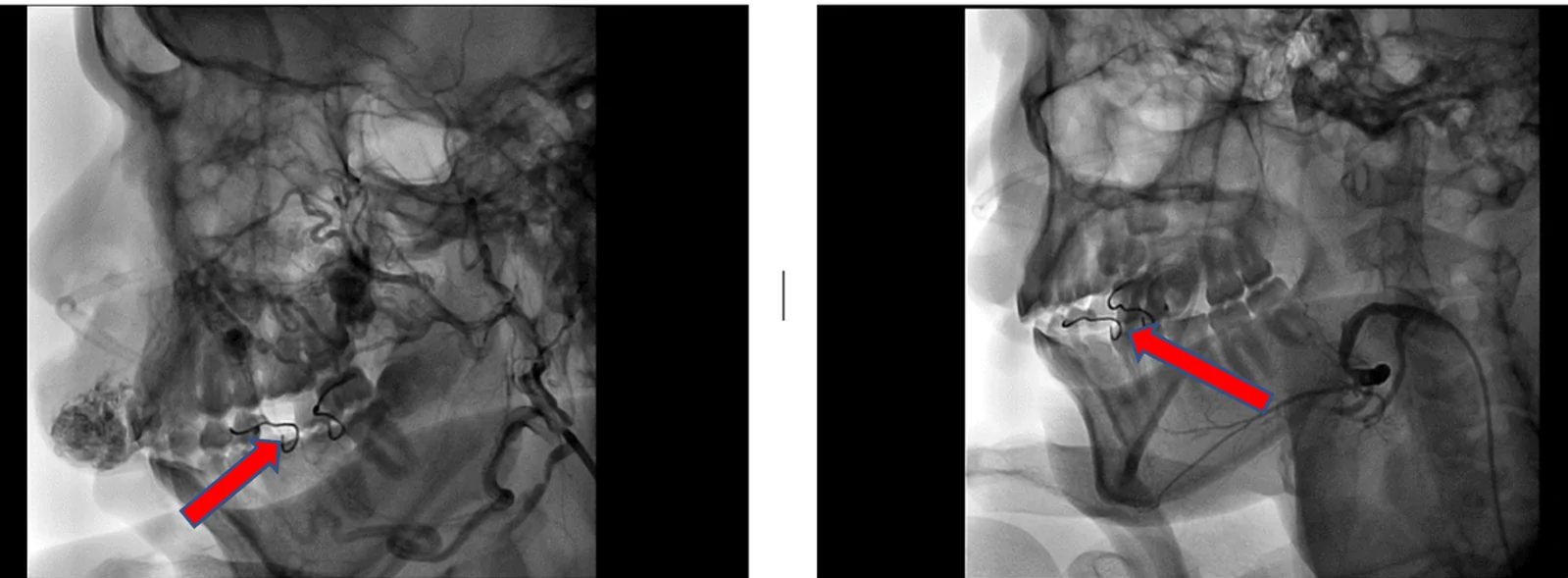
Arteriographic images demonstrating two serpiginous, mobile intravascular structures within the left upper-lip vascular territory, interpreted as suspected filarial worms. (a) Arteriogram showing a serpiginous filling defect (arrow) within the vascular channels of the left upper lip, which changed position during angiography. (b) Corresponding projection showing the same structure in a different position, demonstrating its mobility during angiography. The mobility and serpiginous appearance of these structures, together with the patient’s previous history of filarial infection, were considered suggestive of intravascular filarial involvement.

The patient requested therapeutic intervention for the labial aneurysm due to the aesthetic discomfort it had caused. In June 2015, an arteriography was performed with the intention of embolizing the feeding arteries of the left upper lip aneurysm using coils to minimize intraoperative blood loss during aneurysm resection.

Unexpectedly, during angiography while preparing for coil embolization, two serpiginous, mobile intravascular structures were visualized, changing position during the examination and interpreted as suspected filarial worms in the context of the patient’s previous history of filarial infection (Figure 1). In view of these findings, the planned embolization was discontinued.

Post-angiography re-evaluation of the patient’s history revealed a previous filarial infection during childhood, which had been appropriately treated at that time. Given this history and the angiographic findings, persistent or recurrent infection was considered; however, the available clinical information did not allow definitive differentiation between persistent infection, recurrence, or reinfection, and additional antiparasitic therapy was initiated. The patient subsequently returned to his home country for antiparasitic treatment. The patient received oral ivermectin (200 μg/kg for 12 days) and oral albendazole (400 mg as a single dose). The left labial aneurysm was subsequently resected in January 2017.

In the second stage, a facial CT angiography was performed to evaluate the anatomical relationship between the vascular lesion and adjacent structures in the left cheek and temporal areas (Figure 2). The lesion measured approximately 4 × 4 cm and was characterized by tortuous vascular structures with surrounding dilated vessels, consistent with an arteriovenous pseudoaneurysmal lesion.

**Figure 2:**
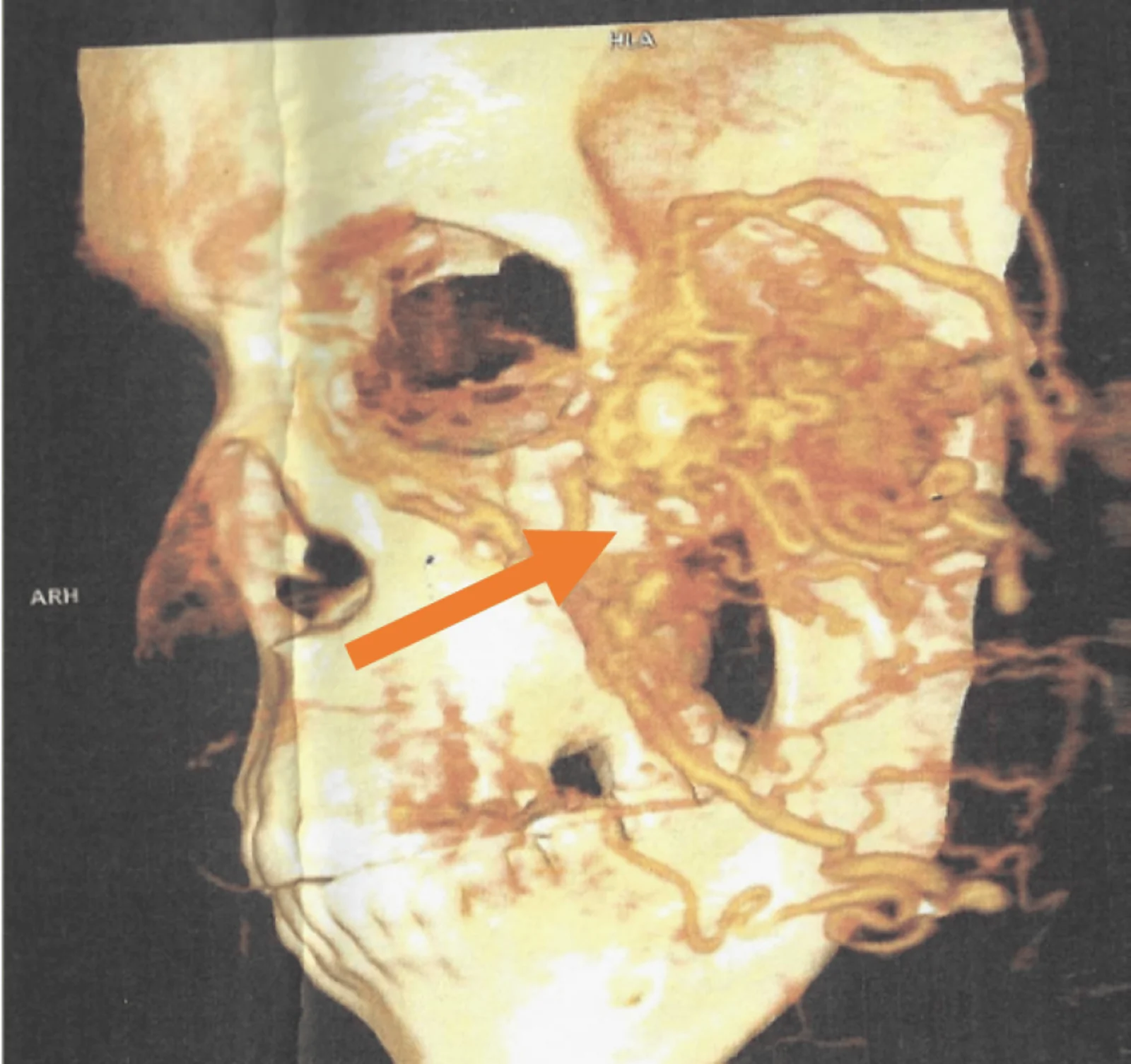
CT angiography showing an arteriovenous pseudoaneurysmal lesion in the left cheek/temporal region. Three-dimensional reconstructed CT scan depicting a vascular abnormality (arrow) in the left cheek, consistent with an arteriovenous pseudoaneurysmal lesion. The image highlights the tortuous, dilated vessels surrounding the lesion, providing anatomical information for surgical planning.

Due to the patient’s professional commitments and limited availability for travel, definitive surgical management of the left cheek/temporal lesion was delayed until September 2023, as the lesion was not causing significant aesthetic concern and had not shown noticeable progression in size.

Complete macroscopic surgical resection of the arteriovenous pseudoaneurysmal lesion was then performed. The lesion was excised while preserving the underlying muscular structures, with no postoperative facial nerve deficit.

Histopathological examination of the resected left cheek/temporal lesion revealed a vascular proliferation composed of large, ectatic, tortuous, and congested vessels, some of which were interconnected. The vessel walls were variably altered: in some areas, they appeared thin and lined with flattened, regular endothelial cells; in others, they were remodeled by early fibrosis associated with a mononuclear inflammatory infiltrate. The vascular proliferation extended between the striated muscle bundles. No filarial organisms were identified in the resected specimen (Figure 3). The patient had received antiparasitic treatment before surgical resection.

**Figure 3:**
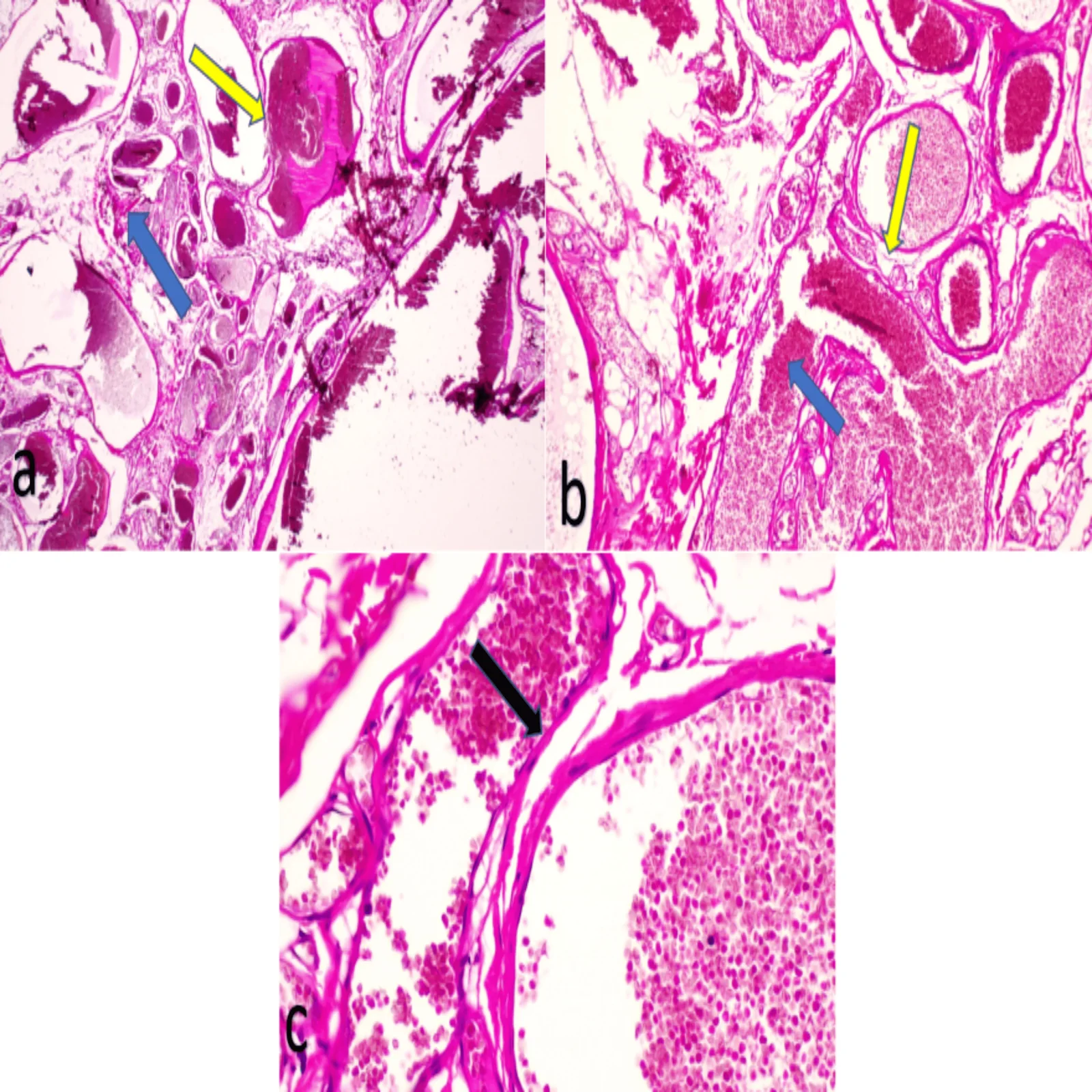
Histopathological findings of the resected left temporal vascular lesion. (a) Low-power view (hematoxylin and eosin (H&E), ×40) showing a vascular proliferation composed of ectatic, tortuous, and congested vessels of variable caliber (arrows). (b) Intermediate-power view (H&E, ×100) demonstrating dilated and congested vascular channels of different sizes (arrows). (c) High-power view (H&E, ×400) showing a vascular channel lined by flattened endothelial cells (black arrow), with erythrocytes within the lumen.

The patient was discharged four days after surgery without pain, paresthesia, or neurological deficits. At the six-month clinical follow-up, he remained asymptomatic, with no reported pain, paresthesia, neurological deficit, or signs of local infection, and had a satisfactory clinical outcome.

## Discussion

Infectious aneurysms, also known as mycotic aneurysms, result from infections that weaken the vascular wall and promote aneurysmal formation. Although the term originally described the fungus-like appearance of these lesions, mycotic aneurysms are most commonly associated with bacterial infections [4]. An aneurysm may arise in the context of systemic infection with bacteremia or through direct local invasion of the vascular wall. Traumatic pseudoaneurysms, by contrast, can occur following vascular injuries caused by physical trauma [5].

The aorta, intracranial arteries, and peripheral and visceral arteries, including the femoral, superior mesenteric, and splenic arteries, may be affected [4]. The natural course of infectious aneurysms may involve progressive expansion and rupture, leading to massive hemorrhage, sepsis, and multiorgan failure. Their high perioperative mortality makes them a significant challenge for vascular surgeons [4].

The mechanisms underlying aneurysm formation in the context of filarial infection remain poorly understood, although rare cases of aneurysms attributed to filarial infection have been reported [1]. Lymphatic filariasis is a parasitic disease transmitted by infected mosquitoes, primarily of the genera *Culex*, *Anopheles*, *Aedes*, and *Mansonia*, and caused primarily by *Wuchereria bancrofti* and *Brugia* species. Adult worms inhabit the lymphatic system, where they can impair lymphatic function and cause manifestations such as lymphedema and elephantiasis [2]. Treatment and prevention strategies depend on the specific filarial infection and epidemiological setting. Antiparasitic regimens may include diethylcarbamazine, ivermectin, and albendazole, while preventive measures include mosquito control and protection against mosquito bites [2].

Aneurysms associated with filarial infection are exceptionally rare, and the available literature is insufficient to establish their natural history, risk of rupture, recurrence rates, or optimal management. Consequently, treatment decisions must be individualized according to the lesion’s anatomical location, clinical presentation, and potential complications. Facial artery aneurysms are also rare, with only a limited number of cases reported in the literature [5]. Further case reports may help improve understanding of these unusual vascular presentations and inform clinical management.

The diagnosis of facial aneurysms relies on clinical examination and imaging. Color Doppler ultrasonography can help identify the lesion and assess blood flow, while CT angiography provides information about vascular anatomy and supports treatment planning [5,6]. Conventional angiography can further characterize the lesion and its feeding vessels [7].

Although diagnostic advances have been reported for parasitic vascular disease in veterinary medicine, including serological approaches based on indirect enzyme-linked immunosorbent assay for *Strongylus vulgaris* infection in horses, these methods have not been validated for diagnosing human filarial vascular disease and therefore cannot currently be extrapolated to the present case [8].

Toledo et al. reported an aneurysm attributed to *Wuchereria bancrofti*, providing an important precedent for a possible association between filarial infection and aneurysmal disease [1]. In our patient, angiography revealed mobile serpiginous intravascular structures that raised suspicion of filarial involvement. However, histopathological examination of the resected lesion demonstrated vascular ectasia, tortuosity, and mononuclear inflammatory infiltration without identifying filarial organisms. Thus, although both cases raise the possibility of a relationship between filarial infection and aneurysmal disease, the evidence for parasitic involvement differs. Nevertheless, the absence of identifiable filarial organisms on histopathological examination does not necessarily exclude a parasitic etiology, particularly considering the visualization of mobile serpiginous intravascular structures on angiography, the patient’s previous history of filarial infection, and the administration of antiparasitic treatment before surgical resection.

Treatment options for facial artery aneurysms include surgical excision, ligation, and endovascular embolization. The choice depends on the lesion’s location and anatomy, the risk of bleeding, and the feasibility of each approach. Surgical excision allows direct control of the lesion but carries risks such as hemorrhage and facial nerve injury. Endovascular embolization offers a less invasive alternative; however, incomplete occlusion or recanalization may occur, and follow-up imaging may be necessary [5,9,10].

In our case, coil embolization was initially planned to reduce the risk of intraoperative bleeding but was discontinued after mobile serpiginous intravascular structures suggestive of filarial involvement were observed during angiography. The patient subsequently received antiparasitic treatment and underwent surgical resection. Nakagawa et al. reported the use of internal trapping followed by surgical excision to manage a facial artery aneurysm and reduce intraoperative bleeding [7]. Although combined endovascular and surgical approaches may be useful in selected cases, evidence specific to suspected verminous aneurysms remains extremely limited.

## Conclusions

Verminous aneurysm is an exceptionally rare vascular manifestation potentially associated with parasitic infection and may present with diagnostic and therapeutic challenges. This case of a facial vascular lesion in a patient with a previous history of filarial infection highlights the diagnostic challenges associated with suspected parasitic vascular involvement. Although filarial involvement was suspected based on the patient’s history and angiographic findings, no filarial organisms were identified on histopathological examination. A careful diagnostic approach and individualized management are essential in such rare presentations. Further reported cases are needed to better understand their pathophysiology and establish optimal diagnostic and therapeutic strategies.
